# Tetra­carbonyl-1κ^2^ *C*,3κ^2^ *C*-bis[1,3(η^5^)-cyclo­penta­dien­yl]dihydroxido-2κ^2^ *O*-diirontin(2 *Fe—Sn*) monohydrate

**DOI:** 10.1107/S1600536810021975

**Published:** 2010-06-18

**Authors:** Stefanie Kössel, Christoph Wagner, Kurt Merzweiler

**Affiliations:** a Institut für Chemie, Naturwissenschaftliche Fakulät II, Martin-Luther-Universität Halle-Wittenberg, Kurt-Mothes-Strasse 2, 06120 Halle, Germany

## Abstract

In the title hydrate, [Fe_2_Sn(C_5_H_5_)_2_(OH)_2_(CO)_4_]·H_2_O, the central Sn atom is tetra­hedrally coordinated by two {Cp(CO)_2_Fe} fragments and two hydroxide groups. The [{Cp(CO)_2_Fe}_2_Sn(OH)_2_] and water mol­ecules are linked by O—H⋯O hydrogen bridges, giving two-dimensional arrays with 4.8^2^ topology that stack along the *c* axis.

## Related literature

For the crystal structures of diorganotin dihydroxides, see: Pu *et al.* (2001 ▶); Tajima *et al.* (2006 ▶). For the related structure of [{Cp(CO)_2_Fe}_2_Sn(OH)_2_] (without experimental details), see: Nesmeyanov *et al.* (1966 ▶). For related structures [{Cp(CO)_2_Fe}_3_SnOH, see: O’Connor & Corey (1967 ▶); Fässler & Schütz (1997 ▶).
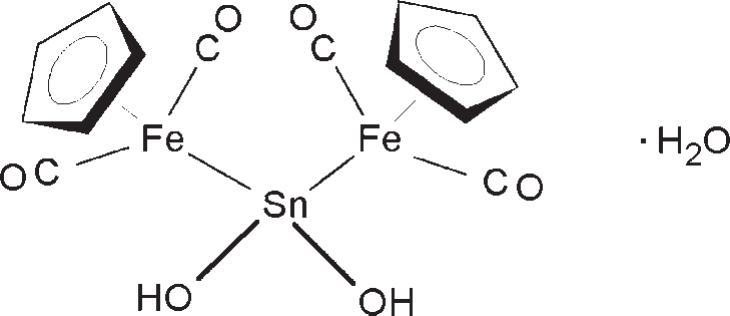


## Experimental

### 

#### Crystal data

[Fe_2_Sn(C_5_H_5_)_2_(OH)_2_(CO)_4_]·H_2_O
*M* *_r_* = 524.64
Triclinic, 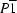

*a* = 7.1760 (6) Å
*b* = 9.7262 (9) Å
*c* = 12.063 (1) Å
α = 92.046 (7)°
β = 90.822 (7)°
γ = 97.560 (7)°
*V* = 833.93 (12) Å^3^
*Z* = 2
Mo *K*α radiation
μ = 3.24 mm^−1^
*T* = 200 K
0.16 × 0.15 × 0.07 mm

#### Data collection

Stoe IPDS 2T diffractometer
Absorption correction: numerical (*X-AREA*; Stoe & Cie, 2009 ▶) *T*_min_ = 0.607, *T*_max_ = 0.806
7904 measured reflections
3641 independent reflections
3374 reflections with *I* > 2σ(*I*)
*R*_int_ = 0.018

#### Refinement

*R*[*F* ^2^ > 2σ(*F* ^2^)] = 0.019
*wR*(*F* ^2^) = 0.048
*S* = 1.05
3641 reflections
233 parameters
4 restraints
H atoms treated by a mixture of independent and constrained refinement
Δρ_max_ = 0.44 e Å^−3^
Δρ_min_ = −0.62 e Å^−3^

### 

Data collection: *X-AREA* (Stoe & Cie, 2009 ▶); cell refinement: *X-AREA*; data reduction: *X-AREA*; program(s) used to solve structure: *SHELXS97* (Sheldrick, 2008 ▶); program(s) used to refine structure: *SHELXL97* (Sheldrick, 2008 ▶); molecular graphics: *DIAMOND* (Brandenburg, 2009 ▶); software used to prepare material for publication: *SHELXL97* and *PLATON* (Spek, 2009 ▶).

## Supplementary Material

Crystal structure: contains datablocks I, global. DOI: 10.1107/S1600536810021975/tk2678sup1.cif

Structure factors: contains datablocks I. DOI: 10.1107/S1600536810021975/tk2678Isup2.hkl

Additional supplementary materials:  crystallographic information; 3D view; checkCIF report

## Figures and Tables

**Table 1:** Hydrogen-bond geometry (Å, °)

*D*—H⋯*A*	*D*—H	H⋯*A*	*D*⋯*A*	*D*—H⋯*A*
O5—H1⋯O7^i^	0.80 (4)	2.01 (4)	2.778 (2)	159 (4)
O6—H2⋯O1^ii^	0.80 (3)	2.44 (3)	3.212 (2)	160 (3)
O7—H3⋯O5^iii^	0.82 (4)	1.96 (4)	2.773 (3)	176 (4)
O7—H4⋯O6	0.82 (3)	1.90 (3)	2.709 (2)	174 (4)

Symmetry codes: (i) 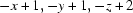; (ii) 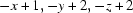; (iii) 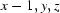.

**Fig. 1.**
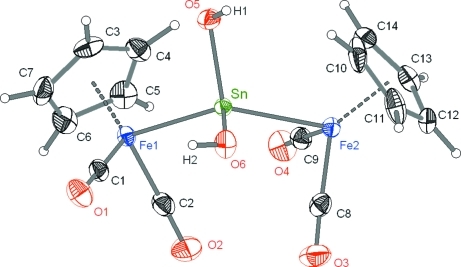
Molecular structure of (I) showing displacement ellipsoids at the 40 % probability level.

**Fig. 2.**
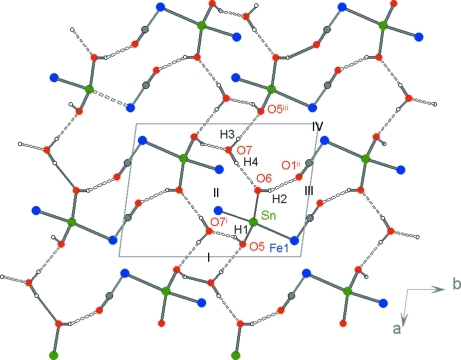
Part of the hydrogen bond network of (I) exhibiting 4.82 topology (Cp groups and CO groups not involved in hydrogen bonding are omitted for clarity).

## supplementary crystallographic information

### Comment

In the structure of the title compound, (I), a known material but with limited characterisation data (Nesmeyanov *et al.*, 1966), the tin atom is surrounded by two {Cp(CO)_2_Fe} fragments and two OH groups in a distorted tetrahedral arrangement (Fig. 1). The {Cp(CO)_2_Fe}_2_Sn unit shows experimentally equivalent Sn—Fe bond lengths of 2.534 (1) and 2.535 (1) Å, and a Fe—Sn—Fe angle of 122.8 (1) °. Compared to [{Cp(CO)_2_Fe}_2_SnCl_2_] (Sn—Fe: 2.492 (8) Å; Fe—Sn—Fe: 128.6 (3) °), the Sn—Fe distances are around 0.05 Å longer and the Fe—Sn—Fe angle is reduced by 6° (O'Connor & Corey, 1967). Compound (I) also contains two Sn—O bonds of 2.016 (2) and 2.026 (2) Å which are close to the Sn—O distance found in [{Cp(CO)_2_Fe}_3_SnOH] (Fässler & Schütz, 1997). As compared to the dihydroxy derivatives (2,6-Mes_2_H_3_C_6_)_2_Sn(OH)_2_ (Sn—O: 1.97 (4) Å ) (Pu *et al.*, 2001) and (Bbt)(Titp)Sn(OH)_2_ (Sn—O: 1.990 (4) and 2.005 (4) Å) ((Bbt = 2,6-{(CH(SiMe_3_)_2_}_2_-4-{CSiMe_3_}_3_H_2_C_6_; Titp = 2,6-{2,4-(i-Pr)_2_H_3_C_6_}H_4_C_6_) (Tajima *et al.*, 2006), the Sn—O bonds in (I) are slightly enlongated. This might be due to the presence of hydrogen bonds involving both OH groups but in different types of hydrogen bridges, Fig. 2. Two hydrogen bridges are formed between the O7-water molecule as donator and the hydroxyl oxygen atoms O5^iii^ and O6 as acceptors. Additionally, the hydroxyl group O5–H1 forms a hydrogen bridge to the water oxygen atom O7^i^ and the hydroxyl group O6–H2 is involved in a hydrogen bridge with the carbonyl oxygen atom O1^ii^. Consequently, a two-dimensional array is formed that comprises 8-membered O_4_H_4_ rings (I in Fig. 2), 12-membered Sn_2_O_6_H_4_ rings (II), 12-membered Sn_2_Fe_2_C_2_O_4_H_2_ rings (III) and 20-membered Sn_2_Fe_2_C_2_O_8_H_6_ rings (IV). The interconnection of theses rings leads to a three-connected net with 4.8^2 ^topology.

### Experimental

To a solution of 3.24 g (6.2 mmol) [{Cp(CO)_2_Fe}_2_SnCl_2_] in CH_2_Cl_2_ (15 ml), a solution of NaOH (2.4 g, 60 mmol) in water (9 ml) was added at 273 K. Within 3 h, yellow-orange crystals of (I) were formed at the CH_2_Cl_2_/H_2_O interface. The precipitate was filtered, washed with water, and dried. Yield: 2.37 g (73 %). Anal. Calcd. for C_14_H_14_Fe_2_O_7_Sn (524.64): C 32.05, H 2.69 %. Found: C 31.33, H 2.61 %. ^1^H-NMR (methanol-d_4_): d = 5.11 (s, 10 H, Cp) p.p.m. ^13^C-NMR (methanol-d_4_): d = 84.3 (Cp), 214.0 (CO) p.p.m. ^119^Sn-NMR (methanol-d_4_): d = 454 (s) p.p.m.

### Refinement

Whereas the O-bound H atoms were refined freely, the C-bound H atoms were geometrically placed (C-H = 0.95 Å) and refined as riding with *U*_iso_(H) = 1.2*U*_eq_(C).

### Crystal data

**Table:**

[Fe_2_Sn(C_5_H_5_)_2_(OH)_2_(CO)_4_]·H_2_O	*Z* = 2
*M**_r_* = 524.64	*F*(000) = 512
Triclinic, *P*1	*D*_x_ = 2.089 Mg m^−^^3^
Hall symbol: -P 1	Mo *K*α radiation, λ = 0.71073 Å
*a* = 7.1760 (6) Å	Cell parameters from 8934 reflections
*b* = 9.7262 (9) Å	θ = 5.3–53.6°
*c* = 12.063 (1) Å	µ = 3.24 mm^−^^1^
α = 92.046 (7)°	*T* = 200 K
β = 90.822 (7)°	Prism, orange
γ = 97.560 (7)°	0.16 × 0.15 × 0.07 mm
*V* = 833.93 (12) Å^3^	

### Data collection

**Table:**

Stoe IPDS 2T diffractometer	3641 independent reflections
Radiation source: fine-focus sealed tube	3374 reflections with *I* > 2σ(*I*)
graphite	*R*_int_ = 0.018
Detector resolution: 6.67 pixels mm^-1^	θ_max_ = 27.0°, θ_min_ = 2.7°
rotation method scans	*h* = −9→9
Absorption correction: numerical (*X-AREA*; Stoe & Cie, 2009)	*k* = −12→11
*T*_min_ = 0.607, *T*_max_ = 0.806	*l* = −15→15
7904 measured reflections	

### Refinement

**Table:**

Refinement on *F*^2^	Primary atom site location: structure-invariant direct methods
Least-squares matrix: full	Secondary atom site location: difference Fourier map
*R*[*F*^2^ > 2σ(*F*^2^)] = 0.019	Hydrogen site location: inferred from neighbouring sites
*wR*(*F*^2^) = 0.048	H atoms treated by a mixture of independent and constrained refinement
*S* = 1.05	*w* = 1/[σ^2^(*F*_o_^2^) + (0.0325*P*)^2^ + 0.1254*P*] where *P* = (*F*_o_^2^ + 2*F*_c_^2^)/3
3641 reflections	(Δ/σ)_max_ = 0.002
233 parameters	Δρ_max_ = 0.44 e Å^−^^3^
4 restraints	Δρ_min_ = −0.62 e Å^−^^3^
0 constraints	

### Special details

**Table:**

Geometry. All esds (except the esd in the dihedral angle between two l.s. planes) are estimated using the full covariance matrix. The cell esds are taken into account individually in the estimation of esds in distances, angles and torsion angles; correlations between esds in cell parameters are only used when they are defined by crystal symmetry. An approximate (isotropic) treatment of cell esds is used for estimating esds involving l.s. planes.
Refinement. Refinement of *F*^2^ against ALL reflections. The weighted *R*-factor wR and goodness of fit *S* are based on *F*^2^, conventional *R*-factors *R* are based on *F*, with *F* set to zero for negative *F*^2^. The threshold expression of *F*^2^ > σ(*F*^2^) is used only for calculating *R*-factors(gt) etc. and is not relevant to the choice of reflections for refinement. *R*-factors based on *F*^2^ are statistically about twice as large as those based on *F*, and *R*- factors based on ALL data will be even larger.

### Fractional atomic coordinates and isotropic or equivalent isotropic displacement parameters (Å^2^)

**Table:**

	*x*	*y*	*z*	*U*_iso_*/*U*_eq_	
C1	0.7075 (3)	1.0222 (2)	0.84442 (18)	0.0283 (4)	
C2	0.7280 (4)	0.9336 (2)	0.64429 (19)	0.0350 (5)	
C3	1.1193 (3)	0.9698 (3)	0.8631 (2)	0.0393 (5)	
H3A	1.1195	0.9477	0.9384	0.047*	
C4	1.1382 (3)	0.8774 (3)	0.7722 (2)	0.0367 (5)	
H4A	1.1547	0.7834	0.7761	0.044*	
C5	1.1278 (3)	0.9515 (3)	0.6750 (2)	0.0395 (5)	
H5A	1.1349	0.9151	0.6022	0.047*	
C6	1.1047 (3)	1.0908 (3)	0.7051 (2)	0.0418 (6)	
H6A	1.0946	1.1626	0.6561	0.050*	
C7	1.0999 (3)	1.1017 (3)	0.8212 (2)	0.0418 (6)	
H7A	1.0861	1.1824	0.8638	0.050*	
C8	0.4498 (3)	0.5867 (2)	0.65282 (18)	0.0318 (5)	
C9	0.7911 (3)	0.6018 (2)	0.58481 (18)	0.0298 (4)	
C10	0.6902 (6)	0.3898 (3)	0.8208 (2)	0.0596 (10)	
H10A	0.7103	0.4226	0.8948	0.072*	
C11	0.5143 (4)	0.3457 (3)	0.7690 (2)	0.0482 (7)	
H11A	0.3964	0.3455	0.8019	0.058*	
C12	0.5462 (3)	0.3020 (2)	0.6590 (2)	0.0356 (5)	
H12A	0.4534	0.2666	0.6059	0.043*	
C13	0.7395 (4)	0.3204 (2)	0.6431 (2)	0.0378 (5)	
H13A	0.7997	0.2992	0.5771	0.045*	
C14	0.8301 (4)	0.3761 (3)	0.7426 (3)	0.0509 (8)	
H14A	0.9604	0.3997	0.7543	0.061*	
O1	0.5976 (3)	1.06847 (19)	0.89760 (16)	0.0420 (4)	
O2	0.6339 (3)	0.9215 (2)	0.56601 (17)	0.0582 (6)	
O3	0.3141 (3)	0.6314 (2)	0.63030 (16)	0.0492 (5)	
O4	0.8793 (3)	0.6577 (2)	0.51681 (15)	0.0450 (4)	
O5	0.9048 (2)	0.67485 (17)	0.94997 (13)	0.0328 (3)	
H1	0.851 (5)	0.625 (4)	0.994 (3)	0.080 (13)*	
O6	0.5023 (2)	0.71819 (18)	0.90976 (14)	0.0353 (4)	
H2	0.502 (4)	0.783 (3)	0.953 (2)	0.049 (9)*	
O7	0.1945 (2)	0.5242 (2)	0.89430 (15)	0.0376 (4)	
H4	0.292 (3)	0.578 (3)	0.901 (3)	0.057 (10)*	
H3	0.112 (5)	0.572 (4)	0.910 (4)	0.090 (15)*	
Sn1	0.740305 (17)	0.716117 (13)	0.821699 (10)	0.02046 (5)	
Fe1	0.87665 (4)	0.95526 (3)	0.76220 (2)	0.02269 (7)	
Fe2	0.65366 (4)	0.51278 (3)	0.68520 (2)	0.02259 (7)	

### Atomic displacement parameters (Å^2^)

**Table:**

	*U*^11^	*U*^22^	*U*^33^	*U*^12^	*U*^13^	*U*^23^
C1	0.0331 (11)	0.0212 (9)	0.0303 (10)	0.0017 (8)	0.0000 (8)	0.0036 (8)
C2	0.0458 (13)	0.0272 (11)	0.0330 (12)	0.0079 (10)	−0.0006 (10)	0.0060 (9)
C3	0.0238 (10)	0.0580 (16)	0.0360 (12)	0.0042 (10)	−0.0036 (9)	0.0052 (11)
C4	0.0243 (10)	0.0338 (12)	0.0533 (14)	0.0064 (9)	0.0080 (9)	0.0078 (10)
C5	0.0337 (12)	0.0454 (14)	0.0392 (13)	0.0034 (10)	0.0146 (10)	0.0015 (10)
C6	0.0324 (12)	0.0335 (12)	0.0581 (16)	−0.0056 (10)	0.0098 (11)	0.0155 (11)
C7	0.0286 (11)	0.0352 (13)	0.0580 (16)	−0.0055 (10)	0.0021 (10)	−0.0119 (11)
C8	0.0340 (11)	0.0346 (12)	0.0260 (10)	0.0036 (9)	−0.0002 (8)	−0.0065 (8)
C9	0.0341 (11)	0.0287 (11)	0.0274 (10)	0.0079 (9)	0.0007 (8)	−0.0018 (8)
C10	0.124 (3)	0.0198 (11)	0.0302 (12)	−0.0077 (14)	−0.0171 (15)	0.0043 (9)
C11	0.0671 (18)	0.0231 (11)	0.0522 (15)	−0.0056 (11)	0.0291 (14)	0.0042 (10)
C12	0.0409 (12)	0.0214 (10)	0.0424 (13)	−0.0020 (9)	−0.0056 (10)	−0.0036 (9)
C13	0.0450 (13)	0.0246 (11)	0.0454 (13)	0.0110 (10)	0.0065 (10)	0.0010 (9)
C14	0.0486 (15)	0.0267 (12)	0.077 (2)	0.0026 (11)	−0.0287 (15)	0.0095 (12)
O1	0.0448 (10)	0.0374 (9)	0.0466 (10)	0.0143 (8)	0.0139 (8)	0.0006 (8)
O2	0.0768 (15)	0.0588 (13)	0.0399 (11)	0.0149 (11)	−0.0259 (10)	0.0021 (9)
O3	0.0404 (10)	0.0665 (13)	0.0441 (10)	0.0240 (9)	−0.0098 (8)	−0.0100 (9)
O4	0.0508 (11)	0.0462 (10)	0.0377 (9)	0.0021 (8)	0.0165 (8)	0.0090 (8)
O5	0.0357 (8)	0.0326 (8)	0.0294 (8)	0.0022 (7)	−0.0083 (6)	0.0059 (6)
O6	0.0304 (8)	0.0329 (9)	0.0414 (9)	0.0003 (7)	0.0125 (7)	−0.0051 (7)
O7	0.0282 (8)	0.0444 (10)	0.0398 (9)	0.0015 (8)	0.0035 (7)	0.0093 (8)
Sn1	0.02214 (7)	0.01840 (7)	0.02058 (7)	0.00165 (5)	0.00004 (5)	0.00084 (5)
Fe1	0.02485 (14)	0.01989 (14)	0.02322 (14)	0.00213 (11)	0.00141 (11)	0.00217 (10)
Fe2	0.02546 (14)	0.02059 (14)	0.02121 (14)	0.00140 (11)	0.00051 (10)	−0.00018 (10)

### Geometric parameters (Å, °)

**Table:**

C1—O1	1.151 (3)	C10—C14	1.404 (5)
C1—Fe1	1.754 (2)	C10—C11	1.408 (5)
C2—O2	1.147 (3)	C10—Fe2	2.094 (3)
C2—Fe1	1.755 (2)	C10—H10A	0.9500
C3—C4	1.412 (4)	C11—C12	1.409 (4)
C3—C7	1.419 (4)	C11—Fe2	2.093 (2)
C3—Fe1	2.097 (2)	C11—H11A	0.9500
C3—H3A	0.9500	C12—C13	1.393 (3)
C4—C5	1.405 (4)	C12—Fe2	2.105 (2)
C4—Fe1	2.118 (2)	C12—H12A	0.9500
C4—H4A	0.9500	C13—C14	1.413 (4)
C5—C6	1.422 (4)	C13—Fe2	2.094 (2)
C5—Fe1	2.103 (2)	C13—H13A	0.9500
C5—H5A	0.9500	C14—Fe2	2.082 (3)
C6—C7	1.401 (4)	C14—H14A	0.9500
C6—Fe1	2.103 (2)	O5—Sn1	2.0164 (15)
C6—H6A	0.9500	O5—H1	0.80 (4)
C7—Fe1	2.097 (2)	O6—Sn1	2.0264 (15)
C7—H7A	0.9500	O6—H2	0.80 (3)
C8—O3	1.150 (3)	O7—H4	0.82 (4)
C8—Fe2	1.758 (2)	O7—H3	0.82 (3)
C9—O4	1.151 (3)	Sn1—Fe1	2.5344 (4)
C9—Fe2	1.753 (2)	Sn1—Fe2	2.5345 (4)
			
O1—C1—Fe1	178.8 (2)	Sn1—O6—H2	116 (2)
O2—C2—Fe1	178.4 (2)	H4—O7—H3	104 (4)
C4—C3—C7	108.1 (2)	O5—Sn1—O6	96.19 (7)
C4—C3—Fe1	71.22 (14)	O5—Sn1—Fe1	105.28 (5)
C7—C3—Fe1	70.21 (14)	O6—Sn1—Fe1	112.76 (5)
C4—C3—H3A	125.9	O5—Sn1—Fe2	114.09 (5)
C7—C3—H3A	125.9	O6—Sn1—Fe2	102.65 (5)
Fe1—C3—H3A	124.2	Fe1—Sn1—Fe2	122.793 (12)
C5—C4—C3	107.6 (2)	C1—Fe1—C2	93.08 (11)
C5—C4—Fe1	70.02 (14)	C1—Fe1—C7	94.64 (10)
C3—C4—Fe1	69.62 (13)	C2—Fe1—C7	136.18 (11)
C5—C4—H4A	126.2	C1—Fe1—C3	105.40 (10)
C3—C4—H4A	126.2	C2—Fe1—C3	161.02 (11)
Fe1—C4—H4A	125.7	C7—Fe1—C3	39.57 (11)
C4—C5—C6	108.6 (2)	C1—Fe1—C6	119.32 (10)
C4—C5—Fe1	71.11 (13)	C2—Fe1—C6	101.41 (11)
C6—C5—Fe1	70.21 (13)	C7—Fe1—C6	38.99 (11)
C4—C5—H5A	125.7	C3—Fe1—C6	65.91 (11)
C6—C5—H5A	125.7	C1—Fe1—C5	158.47 (10)
Fe1—C5—H5A	124.6	C2—Fe1—C5	95.64 (11)
C7—C6—C5	107.6 (2)	C7—Fe1—C5	65.68 (10)
C7—C6—Fe1	70.27 (13)	C3—Fe1—C5	65.51 (10)
C5—C6—Fe1	70.28 (13)	C6—Fe1—C5	39.51 (10)
C7—C6—H6A	126.2	C1—Fe1—C4	142.15 (10)
C5—C6—H6A	126.2	C2—Fe1—C4	123.75 (11)
Fe1—C6—H6A	124.8	C7—Fe1—C4	65.92 (10)
C6—C7—C3	108.1 (2)	C3—Fe1—C4	39.16 (10)
C6—C7—Fe1	70.73 (14)	C6—Fe1—C4	65.89 (10)
C3—C7—Fe1	70.22 (13)	C5—Fe1—C4	38.87 (10)
C6—C7—H7A	125.9	C1—Fe1—Sn1	87.56 (7)
C3—C7—H7A	125.9	C2—Fe1—Sn1	89.50 (8)
Fe1—C7—H7A	124.7	C7—Fe1—Sn1	133.84 (8)
O3—C8—Fe2	178.0 (2)	C3—Fe1—Sn1	95.50 (8)
O4—C9—Fe2	178.2 (2)	C6—Fe1—Sn1	149.88 (8)
C14—C10—C11	108.0 (2)	C5—Fe1—Sn1	112.11 (8)
C14—C10—Fe2	69.88 (15)	C4—Fe1—Sn1	84.64 (7)
C11—C10—Fe2	70.31 (16)	C9—Fe2—C8	94.65 (11)
C14—C10—H10A	126.0	C9—Fe2—C14	102.33 (12)
C11—C10—H10A	126.0	C8—Fe2—C14	161.44 (12)
Fe2—C10—H10A	125.4	C9—Fe2—C11	159.00 (10)
C10—C11—C12	108.0 (3)	C8—Fe2—C11	95.48 (12)
C10—C11—Fe2	70.39 (14)	C14—Fe2—C11	66.05 (12)
C12—C11—Fe2	70.83 (13)	C9—Fe2—C13	94.25 (10)
C10—C11—H11A	126.0	C8—Fe2—C13	132.50 (10)
C12—C11—H11A	126.0	C14—Fe2—C13	39.55 (11)
Fe2—C11—H11A	124.4	C11—Fe2—C13	65.48 (10)
C13—C12—C11	107.8 (2)	C9—Fe2—C10	138.61 (14)
C13—C12—Fe2	70.21 (13)	C8—Fe2—C10	125.93 (14)
C11—C12—Fe2	69.93 (13)	C14—Fe2—C10	39.28 (14)
C13—C12—H12A	126.1	C11—Fe2—C10	39.30 (13)
C11—C12—H12A	126.1	C13—Fe2—C10	65.69 (11)
Fe2—C12—H12A	125.4	C9—Fe2—C12	120.77 (10)
C12—C13—C14	108.6 (2)	C8—Fe2—C12	98.94 (10)
C12—C13—Fe2	71.05 (13)	C14—Fe2—C12	65.94 (10)
C14—C13—Fe2	69.75 (15)	C11—Fe2—C12	39.23 (10)
C12—C13—H13A	125.7	C13—Fe2—C12	38.74 (10)
C14—C13—H13A	125.7	C10—Fe2—C12	65.76 (10)
Fe2—C13—H13A	125.1	C9—Fe2—Sn1	89.66 (7)
C10—C14—C13	107.5 (2)	C8—Fe2—Sn1	87.25 (7)
C10—C14—Fe2	70.84 (17)	C14—Fe2—Sn1	100.14 (8)
C13—C14—Fe2	70.70 (14)	C11—Fe2—Sn1	109.15 (8)
C10—C14—H14A	126.2	C13—Fe2—Sn1	139.34 (8)
C13—C14—H14A	126.2	C10—Fe2—Sn1	85.09 (7)
Fe2—C14—H14A	123.9	C12—Fe2—Sn1	147.97 (7)
Sn1—O5—H1	114 (3)		
			
C7—C3—C4—C5	−0.8 (3)	Fe2—Sn1—Fe1—C2	37.77 (8)
Fe1—C3—C4—C5	60.01 (17)	O5—Sn1—Fe1—C7	−2.24 (11)
C7—C3—C4—Fe1	−60.82 (16)	O6—Sn1—Fe1—C7	101.40 (11)
C3—C4—C5—C6	0.7 (3)	Fe2—Sn1—Fe1—C7	−135.05 (10)
Fe1—C4—C5—C6	60.48 (17)	O5—Sn1—Fe1—C3	8.91 (9)
C3—C4—C5—Fe1	−59.76 (16)	O6—Sn1—Fe1—C3	112.55 (9)
C4—C5—C6—C7	−0.3 (3)	Fe2—Sn1—Fe1—C3	−123.89 (7)
Fe1—C5—C6—C7	60.69 (17)	O5—Sn1—Fe1—C6	58.28 (17)
C4—C5—C6—Fe1	−61.04 (17)	O6—Sn1—Fe1—C6	161.92 (17)
C5—C6—C7—C3	−0.2 (3)	Fe2—Sn1—Fe1—C6	−74.52 (16)
Fe1—C6—C7—C3	60.54 (16)	O5—Sn1—Fe1—C5	74.68 (10)
C5—C6—C7—Fe1	−60.69 (17)	O6—Sn1—Fe1—C5	178.33 (10)
C4—C3—C7—C6	0.6 (3)	Fe2—Sn1—Fe1—C5	−58.12 (8)
Fe1—C3—C7—C6	−60.86 (17)	O5—Sn1—Fe1—C4	46.59 (9)
C4—C3—C7—Fe1	61.46 (16)	O6—Sn1—Fe1—C4	150.23 (9)
C14—C10—C11—C12	1.2 (3)	Fe2—Sn1—Fe1—C4	−86.21 (7)
Fe2—C10—C11—C12	61.16 (18)	C10—C14—Fe2—C9	161.03 (16)
C14—C10—C11—Fe2	−59.95 (18)	C13—C14—Fe2—C9	−81.63 (17)
C10—C11—C12—C13	−0.6 (3)	C10—C14—Fe2—C8	−43.2 (4)
Fe2—C11—C12—C13	60.25 (17)	C13—C14—Fe2—C8	74.2 (4)
C10—C11—C12—Fe2	−60.88 (17)	C10—C14—Fe2—C11	−37.42 (16)
C11—C12—C13—C14	−0.2 (3)	C13—C14—Fe2—C11	79.92 (17)
Fe2—C12—C13—C14	59.89 (17)	C10—C14—Fe2—C13	−117.3 (2)
C11—C12—C13—Fe2	−60.08 (17)	C13—C14—Fe2—C10	117.3 (2)
C11—C10—C14—C13	−1.3 (3)	C10—C14—Fe2—C12	−80.55 (17)
Fe2—C10—C14—C13	−61.54 (17)	C13—C14—Fe2—C12	36.79 (15)
C11—C10—C14—Fe2	60.22 (19)	C10—C14—Fe2—Sn1	69.15 (15)
C12—C13—C14—C10	0.9 (3)	C13—C14—Fe2—Sn1	−173.52 (14)
Fe2—C13—C14—C10	61.63 (18)	C10—C11—Fe2—C9	97.1 (4)
C12—C13—C14—Fe2	−60.70 (17)	C12—C11—Fe2—C9	−21.1 (4)
C6—C7—Fe1—C1	−132.89 (16)	C10—C11—Fe2—C8	−144.44 (19)
C3—C7—Fe1—C1	108.66 (15)	C12—C11—Fe2—C8	97.44 (17)
C6—C7—Fe1—C2	−33.6 (2)	C10—C11—Fe2—C14	37.40 (18)
C3—C7—Fe1—C2	−152.00 (17)	C12—C11—Fe2—C14	−80.73 (18)
C6—C7—Fe1—C3	118.4 (2)	C10—C11—Fe2—C13	81.0 (2)
C3—C7—Fe1—C6	−118.4 (2)	C12—C11—Fe2—C13	−37.17 (16)
C6—C7—Fe1—C5	38.06 (16)	C12—C11—Fe2—C10	−118.1 (3)
C3—C7—Fe1—C5	−80.38 (16)	C10—C11—Fe2—C12	118.1 (3)
C6—C7—Fe1—C4	80.86 (17)	C10—C11—Fe2—Sn1	−55.45 (19)
C3—C7—Fe1—C4	−37.59 (15)	C12—C11—Fe2—Sn1	−173.58 (14)
C6—C7—Fe1—Sn1	136.03 (14)	C12—C13—Fe2—C9	−136.65 (16)
C3—C7—Fe1—Sn1	17.59 (19)	C14—C13—Fe2—C9	104.26 (18)
C4—C3—Fe1—C1	163.50 (15)	C12—C13—Fe2—C8	−36.4 (2)
C7—C3—Fe1—C1	−78.37 (16)	C14—C13—Fe2—C8	−155.46 (18)
C4—C3—Fe1—C2	−30.2 (4)	C12—C13—Fe2—C14	119.1 (2)
C7—C3—Fe1—C2	88.0 (4)	C12—C13—Fe2—C11	37.63 (16)
C4—C3—Fe1—C7	−118.1 (2)	C14—C13—Fe2—C11	−81.46 (19)
C4—C3—Fe1—C6	−80.82 (16)	C12—C13—Fe2—C10	80.98 (18)
C7—C3—Fe1—C6	37.30 (15)	C14—C13—Fe2—C10	−38.11 (18)
C4—C3—Fe1—C5	−37.30 (15)	C14—C13—Fe2—C12	−119.1 (2)
C7—C3—Fe1—C5	80.83 (16)	C12—C13—Fe2—Sn1	128.91 (14)
C7—C3—Fe1—C4	118.1 (2)	C14—C13—Fe2—Sn1	9.8 (2)
C4—C3—Fe1—Sn1	74.52 (14)	C14—C10—Fe2—C9	−28.7 (2)
C7—C3—Fe1—Sn1	−167.35 (14)	C11—C10—Fe2—C9	−147.46 (18)
C7—C6—Fe1—C1	56.88 (19)	C14—C10—Fe2—C8	164.40 (15)
C5—C6—Fe1—C1	174.86 (16)	C11—C10—Fe2—C8	45.6 (2)
C7—C6—Fe1—C2	157.02 (16)	C11—C10—Fe2—C14	−118.8 (2)
C5—C6—Fe1—C2	−85.00 (17)	C14—C10—Fe2—C11	118.8 (2)
C5—C6—Fe1—C7	118.0 (2)	C14—C10—Fe2—C13	38.37 (15)
C7—C6—Fe1—C3	−37.85 (16)	C11—C10—Fe2—C13	−80.39 (17)
C5—C6—Fe1—C3	80.13 (17)	C14—C10—Fe2—C12	81.04 (16)
C7—C6—Fe1—C5	−118.0 (2)	C11—C10—Fe2—C12	−37.72 (15)
C7—C6—Fe1—C4	−80.93 (17)	C14—C10—Fe2—Sn1	−112.59 (15)
C5—C6—Fe1—C4	37.05 (16)	C11—C10—Fe2—Sn1	128.65 (17)
C7—C6—Fe1—Sn1	−93.7 (2)	C13—C12—Fe2—C9	52.82 (19)
C5—C6—Fe1—Sn1	24.3 (3)	C11—C12—Fe2—C9	171.38 (18)
C4—C5—Fe1—C1	106.5 (3)	C13—C12—Fe2—C8	153.73 (16)
C6—C5—Fe1—C1	−12.3 (4)	C11—C12—Fe2—C8	−87.71 (19)
C4—C5—Fe1—C2	−140.10 (16)	C13—C12—Fe2—C14	−37.55 (17)
C6—C5—Fe1—C2	101.11 (17)	C11—C12—Fe2—C14	81.0 (2)
C4—C5—Fe1—C7	81.22 (17)	C13—C12—Fe2—C11	−118.6 (2)
C6—C5—Fe1—C7	−37.57 (17)	C11—C12—Fe2—C13	118.6 (2)
C4—C5—Fe1—C3	37.57 (16)	C13—C12—Fe2—C10	−80.77 (19)
C6—C5—Fe1—C3	−81.22 (18)	C11—C12—Fe2—C10	37.8 (2)
C4—C5—Fe1—C6	118.8 (2)	C13—C12—Fe2—Sn1	−107.06 (17)
C6—C5—Fe1—C4	−118.8 (2)	C11—C12—Fe2—Sn1	11.5 (2)
C4—C5—Fe1—Sn1	−48.34 (16)	O5—Sn1—Fe2—C9	−112.12 (9)
C6—C5—Fe1—Sn1	−167.13 (14)	O6—Sn1—Fe2—C9	145.09 (9)
C5—C4—Fe1—C1	−145.01 (17)	Fe1—Sn1—Fe2—C9	17.05 (8)
C3—C4—Fe1—C1	−26.5 (2)	O5—Sn1—Fe2—C8	153.20 (9)
C5—C4—Fe1—C2	50.15 (19)	O6—Sn1—Fe2—C8	50.41 (9)
C3—C4—Fe1—C2	168.67 (16)	Fe1—Sn1—Fe2—C8	−77.63 (8)
C5—C4—Fe1—C7	−80.53 (17)	O5—Sn1—Fe2—C14	−9.65 (11)
C3—C4—Fe1—C7	37.98 (16)	O6—Sn1—Fe2—C14	−112.44 (11)
C5—C4—Fe1—C3	−118.5 (2)	Fe1—Sn1—Fe2—C14	119.52 (9)
C5—C4—Fe1—C6	−37.65 (16)	O5—Sn1—Fe2—C11	58.36 (11)
C3—C4—Fe1—C6	80.87 (17)	O6—Sn1—Fe2—C11	−44.43 (11)
C3—C4—Fe1—C5	118.5 (2)	Fe1—Sn1—Fe2—C11	−172.47 (10)
C5—C4—Fe1—Sn1	135.96 (15)	O5—Sn1—Fe2—C13	−15.99 (12)
C3—C4—Fe1—Sn1	−105.53 (14)	O6—Sn1—Fe2—C13	−118.78 (12)
O5—Sn1—Fe1—C1	−96.33 (9)	Fe1—Sn1—Fe2—C13	113.18 (11)
O6—Sn1—Fe1—C1	7.31 (9)	O5—Sn1—Fe2—C10	26.78 (13)
Fe2—Sn1—Fe1—C1	130.86 (7)	O6—Sn1—Fe2—C10	−76.01 (13)
O5—Sn1—Fe1—C2	170.57 (9)	Fe1—Sn1—Fe2—C10	155.95 (12)
O6—Sn1—Fe1—C2	−85.79 (10)		

### Hydrogen-bond geometry (Å, °)

**Table:**

*D*—H···*A*	*D*—H	H···*A*	*D*···*A*	*D*—H···*A*
O5—H1···O7^i^	0.80 (4)	2.01 (4)	2.778 (2)	159 (4)
O6—H2···O1^ii^	0.80 (3)	2.44 (3)	3.212 (2)	160 (3)
O7—H3···O5^iii^	0.82 (4)	1.96 (4)	2.773 (3)	176 (4)
O7—H4···O6	0.82 (3)	1.90 (3)	2.709 (2)	174 (4)

Symmetry codes: (i) −*x*+1, −*y*+1, −*z*+2; (ii) −*x*+1, −*y*+2, −*z*+2; (iii) *x*−1, *y*, *z*.
